# Evaluation of Intra-Host Variants of the Entire Hepatitis B Virus Genome

**DOI:** 10.1371/journal.pone.0025232

**Published:** 2011-09-20

**Authors:** Sumathi Ramachandran, Xiangjun Zhai, Hong Thai, Davis S. Campo, Guoliang Xia, Lilia M. Ganova-Raeva, Jan Drobeniuc, Yury E. Khudyakov

**Affiliations:** 1 Molecular Epidemiology and Bioinformatics Laboratory, Division of Viral Hepatitis, Centers for Disease Control and Prevention, Atlanta, Georgia, United States of America; 2 Jiangsu Centers for Disease Control, Jiangsu, China; Institut Pasteur, France

## Abstract

Genetic analysis of hepatitis B virus (HBV) frequently involves study of intra-host variants, identification of which is commonly achieved using short regions of the HBV genome. However, the use of short sequences significantly limits evaluation of genetic relatedness among HBV strains. Although analysis of HBV complete genomes using genetic cloning has been developed, its application is highly labor intensive and practiced only infrequently. We describe here a novel approach to whole genome (WG) HBV quasispecies analysis based on end-point, limiting-dilution real-time PCR (EPLD-PCR) for amplification of single HBV genome variants, and their subsequent sequencing. EPLD-PCR was used to analyze WG quasispecies from serum samples of patients (n = 38) infected with HBV genotypes A, B, C, D, E and G. Phylogenetic analysis of the EPLD-isolated HBV-WG quasispecies showed the presence of mixed genotypes, recombinant variants and sub-populations of the virus. A critical observation was that HBV-WG consensus sequences obtained by direct sequencing of PCR fragments without EPLD are genetically close, but not always identical to the major HBV variants in the intra-host population, thus indicating that consensus sequences should be judiciously used in genetic analysis. Sequence-based studies of HBV WG quasispecies should afford a more accurate assessment of HBV evolution in various clinical and epidemiological settings.

## Introduction

Molecular characterization of hepatitis B virus (HBV) is critical to the identification of viral factors that affect outcome of infection, progression and severity of disease as well as responses to antiviral suppression. The complex structure of the HBV genome composed of overlapping and non-overlapping open reading frames (ORFs) significantly constrains HBV evolution [1]. Moreover, the rate of mutation varies among the ORFs [2]. As a result, sub-genomic regions do not reflect evolution of the entire genome. However, genetic analysis of HBV usually involves the study of short genomic regions [3], [4], thereby impeding assessments of HBV evolution [5].

Because of its high mutational rate [6], HBV persists as a large set of closely related variants in infected hosts [7]. Analysis of the genetic composition of the intra-host HBV population is particularly critical to evaluating the emergence of vaccine-escape and drug-resistant mutants. Conventional methods fail to detect minority populations of drug-resistant viral quasispecies if they represent less than 25% of the total sample virus population [8], [9], [10]. Detailed study of the dynamics of viral variants persistent in a given host has long been hampered by the poor sensitivity of sequencing methods. Various approaches have been used to detect mutant HBV populations, though seldom at the whole-genome (WG) level [11], [12]. Genetic cloning is a method of choice for quasispecies assessment. However, it is applied to analysis of short genomic fragments such as the reverse transcriptase (RT) domain or a segment of the core ORF [13]. Furthermore, genetic cloning assays for analysis of intra-host viral populations are time-consuming, labor-intensive, costly and unsuitable for screening large numbers of samples.

The advent of new technologies such as ultra-deep sequencing using the 454 GS FLX sequencer, Illumina genome analyzer and SOLiD sequencing have brought about a paradigm shift in virus research [14], [15], [16]. Their use to sequence multiple genetic variants in HIV [17], [18] and the RT domain of HBV [19], [20] has been reported. However, because of the short read-lengths generated by these technologies, their application is limited to analysis of sub-genomic regions. Assembly of WG sequences for individual viral variants is computationally extensive and is still being developed [21]. Therefore, the information on the WG quasispecies that can be currently obtained using these high-throughput sequencing technologies is primarily in the form of consensus WG sequences accompanied with information on the frequency of nucleotides at polymorphic sites.

Here we report on the development and validation of an automated, medium-throughput approach to WG-HBV sequencing. We had earlier found that end-point, limiting-dilution PCR (EPLD-PCR) is efficient for quasispecies evaluation of sub-genomic regions of hepatitis C virus [22] and HBV [23]. We have also compared the post-PCR cloning and EPLD-PCR protocols applied to intra-host HBV populations [24], and showed a superior sensitivity of EPLD-PCR in detecting HBsAg variants present at frequency as low as 0.1% of the total viral population. Accordingly, in this study, EPLD-PCR was adopted for separation and sequencing of individual intra-host HBV WG variants.

## Results

### EPLD-PCR optimization

Using serum samples collected from patients with acute (n = 8) and chronic HBV infection (n = 30), the performance of the EPLD-PCR approach was compared to consensus PCR amplification.

**Figure 1:**
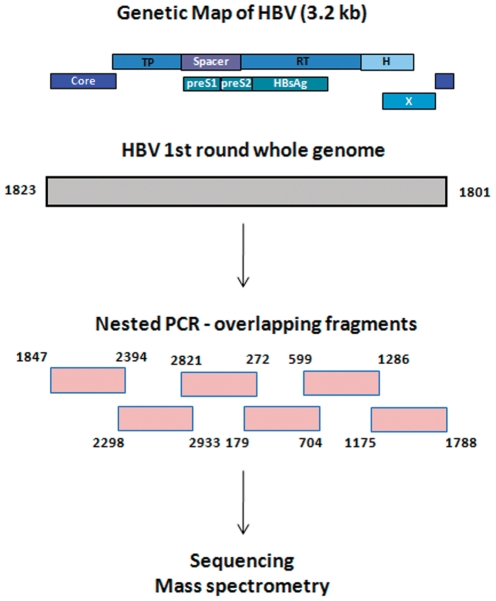
Flowchart of the HBV WG amplification process.

First-round WG amplification was optimized using 4 different primer sets. The primer pair 1801/1823 (WG-F1/R1, Table 1) that amplified HBV genome with a 22-bp gap was a log more sensitive than PCR using the other primer sets, 1798/1801, 1821/1825 and 1849/1855, that amplified the HBV genome with 4, 5 and 7 bp overlap, respectively. If viral titer is not a limitation, the overlap primers could be used for the first round WG amplification, followed by nested PCR with additional two overlapping fragments (F7F/R-1796F/2394R and F8F/R-1176F/1829R). As our study samples included both high- and low-titer samples, we consistently used the 1801/1823 (WG-F1/R1) primer pair, followed by six overlapping nested fragments (F1–F6), for all our analysis (Fig. 1). Detection sensitivity of this approach was 5×10^2^ IU/ml using the World Health Organization (WHO) HBV DNA international standard (NIBSC code: 97/656).

**Table 1:** Primers used for HBV whole genome quasispecies amplification.

Primers	Sequence (5′→3′)	Direction	Nucleotide
**1st round PCR**			
WG-F1	TTTCACCTCTGCCTAATCATCT	Forward	1823
WG-R1	CAGACCAATTTATGCCTACAGCCT	Reverse	1801
WG-F2*	TTCATGTCCTACTGTTCAAGCCTC	Forward	1849
WG-R2*	ACATGAACAAGAGATGATTAGGCAG	Reverse	1855
WG-F3*	TTTTTCACCTCTGCCTAATCATCTC	Forward	1821
WG-R3*	AAAAAGTTGCATGGTGCTGGTGAAC	Reverse	1825
WG-F4*	CTGCGCACCAGCACCATGCAACTTTTTC	Forward	1798
WG-R4*	CAGACCAATTTATGCCTACAGCCT	Reverse	1801
**Nested PCR**			
F1F	TGTTCATGTCCCACTGTTCAA	Forward	1847
F1R	GGCGAGGGAGTTCTT	Reverse	2394
F2F	GACCACCAAATGCCCCTAT	Forward	2298
F2R	TCGGGAAAGAATCCCAGAGGAT	Reverse	2933
F3F	GGTCACCATATTCTTGGGAAC	Forward	2821
F3R	TGAGAGAAGTCCACCACGAGT	Reverse	272
F4F	CTAGGACCCCTGCTCGTGTT	Forward	179
F4R	CGAACCACTGAACAAATGGCACT	Reverse	704
F5F	GTATTCCCATCCCATCATCCTG	Forward	599
F5R	GCTAGGAGTTCCGCAGTATGG	Reverse	1286
F6F	GCCAAGTGTTTGCTGA	Forward	1175
F6R	GCCTACAGCCTCCTA	Reverse	1788
F7F*	GGTCTGCGCACCAGCACCATG	Forward	1796
F7R*	GGCGAGGGAGTTCTT	Reverse	2394
F8F*	GCCAAGTGTTTGCTGAGGCA	Forward	1176
F8R*	TGGTGAAAAAGTTGCATGGTGCTGG	Reverse	1829

**Note**: Each nested primer also contained adaptor sequence tags for sequencing purpose. The forward primers were tagged with SP6 sequence (5′CGATTTAGGTGACACTATAGAAGAGAGGCT3′), and reverse primers with T7 sequence (5′CAGTAATACGACTCACTATAGGGAGAAGGC3′).

*Overlapping WG primers (WG-F2 to F4) when used in the first round PCR, additional two nested primers - F7 and F8 are to be added.

Three different strategies were used to optimize the second-round PCR: namely, (i) two overlapping fragments encompassing the entire genome, (ii) six overlapping fragments of 600 bp each, and (iii) nine overlapping fragments ranging from 350–550 bp. The first set of primers by virtue of its size (>1.5 Kb) was not as sensitive as the other two approaches, and also had the limitation of not being conducive to real-time PCR amplification and sequencing. The nine-PCR-fragment strategy frequently produced incomplete sets of PCR fragments because of differential sensitivity of primers. The six-PCR-fragment approach had a concordant sensitivity among primer sets. Moreover, this strategy produced PCR fragments of lengths that are conducive for the clonal selection process using melt-curve analysis. We therefore used the six primer sets for all WG quasispecies amplification (Table 1, Fig. 1). Additionally, all second-round PCR primers were tagged with sequences of the SP6- or T7-promoters. The application of these tagged primers allowed for the use of only 2 sequencing primers containing SP6 or T7 sequences and for the application of another re-sequencing technology MassArray MALDI-TOF mass spectrometry (Sequenom, Inc., San Diego, CA) in our laboratory [25]. The tagged primers did not compromise the sensitivity of the protocol.

### Validation of single-molecule amplification

For WG quasispecies analysis, EPLD-PCR was performed using serially diluted DNA. At end-point dilution, the DNA target templates are distributed in a Poisson manner so that most reactions (50% or more) do not amplify template molecules and thus do not generate PCR products. The dilution that resulted in positivity in three of six replicates was considered to be limiting, and under such conditions, the positive reactions are most likely to have been initiated from a single-template molecule. We have shown earlier that the function of end-point limiting-dilution in the EPLD-PCR is to limit the number of molecules to a small critical number, from which only one molecule is stochastically amplified in the PCR process [22]. Thus, it is not necessary to dilute specimen down to a single DNA molecule in order to successfully sample single sequence variants. Narrowing down to a minimum pool of DNA molecules prior to PCR amplification is the very important requirement of the technique. However, presence of a pool, though a minimum pool, of HBV DNA molecules at the end-point limiting-dilution generates a theoretical possibility for amplification of more than one molecule during the first-round PCR. In such a scenario, each of six nested PCR fragments has a chance to be amplified from a different first-round WG product, with the assembled WG sequence being composed of a mosaic of fragments derived from different HBV genomes. Therefore, it is important to confirm whether all six fragments amplified by nested PCR are derived from the same, single WG DNA molecule amplified in the first round.

**Figure 2:**
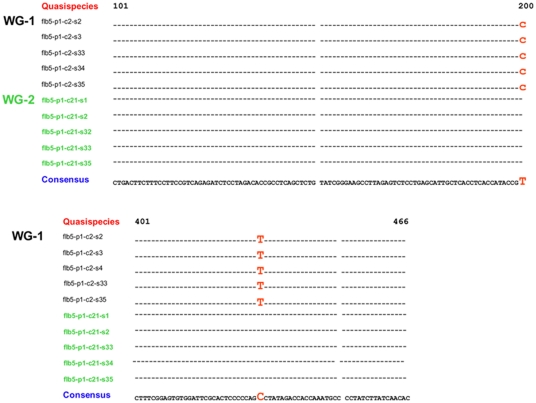
Repeat amplification of the S-gene fragment from first round PCR products. Two WG first-round products (C2 and C21) containing two nucleotide changes (indicated in red) were subjected to 40 independent nested PCR amplifications; a sample of 5 identical S-gene sequences amplified from each clone is shown.

To validate amplification of the final PCR products from a single DNA molecule, we used first-round PCR products obtained from a serum specimen containing two dominant HBV variants, C2 and C21. These variants differed at two nucleotide positions in the HBV *S* gene. These first-round PCR products containing either C2 or C21 were each used to amplify the S gene in 35-40 independent nested PCR reactions. It was expected that the S-gene sequences would belong to both C2 and C21 clones if each of the two first-round products contained a mixture of clones, while these sequences would be identical among all repeats obtained from same first-round products if only a single clone was amplified from a minimum pool of HBV WG DNA molecules at the end-point limiting-dilution. As shown in Fig. 2, all repeats had identical sequences consistent with the corresponding clone, thus strongly confirming that each of the first- and second-round PCR products was indeed amplified from a single C2 or C21 HBV DNA template recovered from the serum specimen. Detection of identical sequences among so many repeats from each first-round PCR product emphasizes the fact that the nucleotide changes observed between C2 and C21 variants are not random PCR errors. The observed high reproducibility of sequences may be attributed to the use of high-fidelity DNA polymerase incorporated in EPLD-PCR, which minimizes the PCR error rate and improves accuracy of DNA amplification.

Since the EPLD-PCR assay requires 5×10^2^ IU/ml of HBV DNA for amplification, this experiment provides an additional proof of concept that the function of end-point limiting-dilution, prior to the amplification process, is to limit the number of DNA molecules to a small critical number, from which only one molecule is then stochastically amplified in the first round PCR (30). The first-round DNA enriched with this one molecule is consequently amplified into overlapping six fragments, which are therefore representative of a single HBV WG variant / template / molecule.

### Effect of viral load

Variation in the number of complete WG clones amplified depends on the viral load of the sample analyzed. For most analyses, we aim at amplifying 50–60 clones per 100 reactions (50% positivity). Fewer clones (<50 clones per 100 reactions) are usually amplified from a sample with a low DNA viral load. On the other hand, extensive end-point limiting-dilution is required to achieve 50% positivity on some samples, which have a high viral load (>10^6^ IU/ml).

Among the 38 serum samples evaluated, about 50–60 clones were amplified from high titer (>10^6^ IU/ml) samples (n = 9), 15–45 clones from intermediate titer (5×10^3^ to 10^6^ IU/ml) samples (n = 15), and 4–14 clones from low titer specimens (n = 10) that have DNA viral load in the range of 5×10^2^ IU/ml (the low end of the WG assay sensitivity). Some low titer specimens tested by EPLD-PCR were obtained after HBe-seroconversion. In very high titer samples (>10^10^ IU/ml, n = 4), clones selected at 50% positivity showed mixed peaks in the nested reaction. Amplicons obtained at that dilution were usually discarded. Selection of clones at 30–35% positivity is recommended for such specimens to avoid mixed sequences. In high titer samples, all clones were usually represented with a complete set of six second-round PCR fragments. However, ∼5–10% of clones failed to generate all six fragments from the low-titer samples. Fragments 1 and 6 were most frequently underrepresented.

### Reproducible detection of intra-host HBV variants

**Figure 3:**
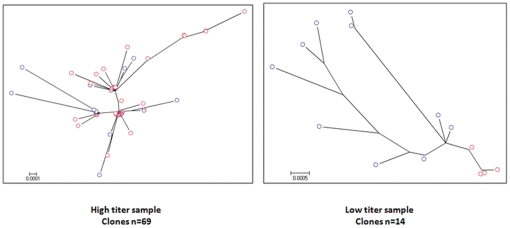
Reproducibility of EPLD protocol for WG-HBV quasispecies amplification. Phylogenetic analysis of HBV-WG quasispecies amplified in two independent batches (red and blue colored nodes) of the same high-titer (a) and low- titer (b) sample.

To assess the reproducibility of assessment of intra-host viral populations, EPLD PCR was repeated twice using 8 samples representing high and low HBV loads. Fig. 3 shows phylogenetic analysis of HBV-WG quasispecies amplified in two independent batches (red and blue colored tips) from one high- and one low-titer sample. A total of 69 clones were amplified from the high-titer sample (Fig. 3a) and 14 from the low-titer specimen (Fig. 3b). As illustrated in Fig. 3, regardless of the number of clones, the quasispecies populations amplified in two independent batches are comparable, and, further, the number of WG clones does not directly correlate with diversity.

### Application to different genotypes

**Figure 4:**
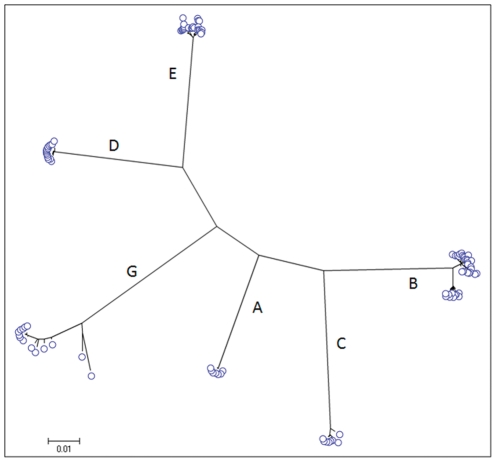
WG-HBV quasispecies amplification of different genotypes. HBV WG was amplified from samples belonging to genotypes A–G using a universal set of primers. Maximum likelihood tree shows WG quasispecies of genotypes A– E and G. Each cluster in the tree is the WG quasispecies of an individual patient belonging to one genotype.

The salient feature of this HBV WG EPLD-PCR approach is that with the help of a single set of universal primers (Table 1), HBV WG quasispecies can be efficiently amplified from major genotypes (A–H). Among the 38 serum samples evaluated for WG quasispecies, we found genotypes A (n = 8), B (n = 3), C (n = 5), D (n = 16), E (n = 3) and G (n = 3). This primer set also amplified HBV WG from specimens of patients infected with HBV genotypes F (n = 2) and H (n = 2). However, because of the low titer of the specimens from these genotypes available to this study, only their consensus WG sequences were obtained. Phylogenetic analyses of the WG quasispecies confirmed genotype classification. Phylogenetic tree of WG quasispecies of six samples belonging to genotypes A–E and G is shown in Fig. 4. The six-fragment WG-assembly was successful in 96–98% of clones for all tested genotypes, suggesting no genotype-specific bias for the universal primers used in this study.

### Detection of mixed genotypes

**Figure 5:**
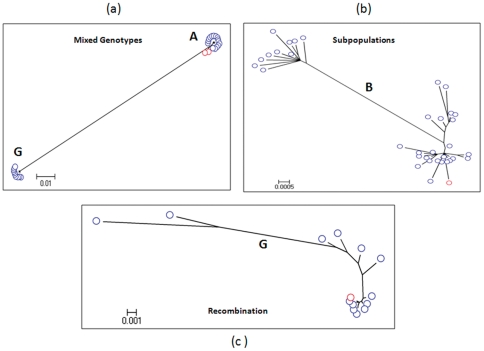
Detection of co-infections, subpopulations and recombination. (**a**) Maximum likelihood trees of HBV-WG quasispecies (blue) and consensus WG sequences from a single patient (viral titer of 10^6^ IU/ml). HBV quasispecies belong to genotypes A and G, while consensus WG sequences (red) obtained at two time-points belong to genotype A. (**b**) Maximum likelihood tree of HBV-WG quasispecies from a genotype B infected patient (viral titer of 10^5^ IU/ml). Consensus sequence shown in red belongs to a single HBV subpopulation, while the WG quasispecies shown in blue segregate into 2 subpopulations. (**c**) Maximum likelihood tree of WG quasispecies (blue) sampled from a patient (viral titer of >10^6^ IU/ml) infected with genotype G and recombinant genotype G/A variants. Consensus sequence (red node) belongs to genotype G. Two HBV WG clones, that are genetically distant from the main cluster of genotype G variants, contain genomic regions belonging to genotype A.

Fig. 5a shows phylogenetic analysis of WG quasispecies from an HBV infected patient. The HBV WG consensus sequence obtained by sequencing of PCR fragments directly amplified from the serum specimen is shown in red, while WG quasispecies obtained by the EPLD-PCR approach are presented as blue. The consensus WG sequence from samples drawn at two time-points from this patient belonged to genotype A. The EPLD-PCR analysis of the quasispecies (blue) from the same patient not only showed a cluster of quasispecies in close proximity to the two consensus sequences, but also co-existence of another cluster belonging to genotype G. It is conceivable that the use of universal primers for consensus sequencing imposed a primer bias for amplification of genotype A over G. However, the use of the genotype G-specific primers for amplification still resulted in the consensus sequence belonging to genotype A (data not shown). Among all tested samples (N = 38), we found mixed genotypes in only two that showed genotype A after consensus WG sequencing, while WG quasispecies belonged to genotypes A and G.

### Detection of intra-host subpopulations

Fig. 5b shows phylogenetic tree of the HBV intra-host WG variants belonging to genotype B (blue). The HBV WG consensus sequence (red) was associated with one viral subpopulation, while EPLD-PCR detected additional WG variants that belonged to two genetically distant viral subpopulations. The consensus sequence for the most part was in close proximity, but not identical to any of the variants including the major variants (Fig. 5a, b and c). The closest sequence to the consensus was at least one nucleotide different.

### Detection of recombination events

Subdivision of HBV quasispecies into subpopulations due to recombination events involving a small fraction of the HBV intra-host population frequently remains unnoticed because of limited capacity of molecular technologies in identifying viral WG quasispecies. Analysis of WG quasispecies from the patient co-infected with genotypes A and G illustrated the capacity of the technique developed in this study to detect and characterize inter-genotypic recombination (Fig. 5c). Among all tested specimens (n = 38), we found this phenomenon only in one. The consensus WG sequence belonging to genotype G (red) revealed no recombination event in the complete-length genome. But a closer examination of the intra-host WG population indicated presence of two variants that were distant from the major subpopulation (blue). SimPlot and bootscan analyses revealed that these two clones were hybrids generated via inter-genotypic recombination between genotypes G and A. One genotype G recombinant variant contained the genotype A sequences at nucleotide position 704–1167 (S and Polymerase region) and the other variant at 2005–2411(Core region). In order to show that the recombination event identified through the EPLD-PCR was not due to PCR introduced errors, we used genotype A and G specific primers on (i) genotype A alone and G alone samples independently, (ii) also physically mixing these 2 genotype samples in different ratios, (iii) specimen shown in Fig 4c, that shows recombination (A and G) and (iv) two other samples which show A and G mixed genotype, but no recombination. We unanimously saw that physical mixing of the two genotypes, or mixed genotypes naturally occurring in an infection, did not necessarily give rise to any recombination due to the PCR process.

### Distribution of polymorphic sites along the genome

**Figure 6:**
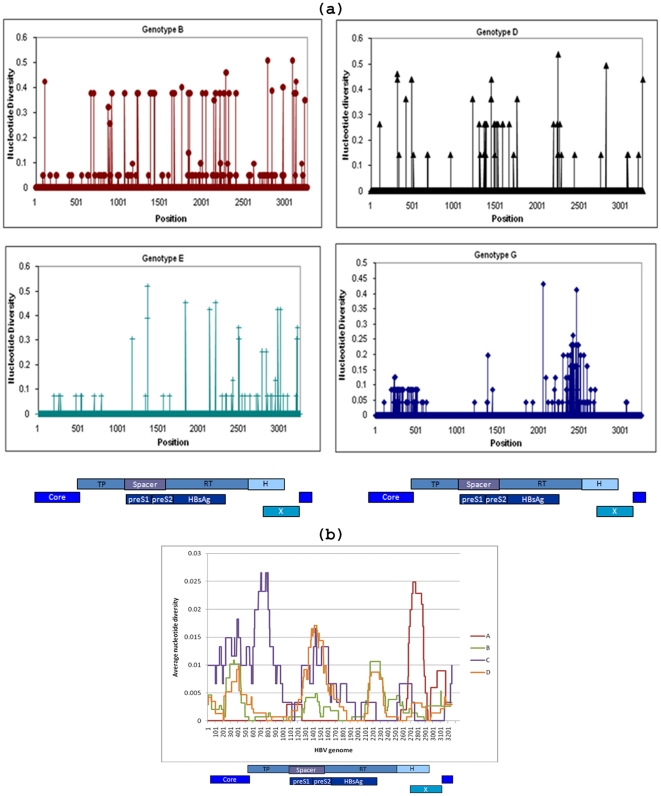
Distribution of mutations along the HBV genome. (**a**) Polymorphic positions along the HBV WG quasispecies obtained from 4 patients infected with different HBV genotypes (viral titer of 10^3^–10^6^ IU/ml). (**b**) Sliding window analysis of polymorphic regions identified among genotype D variants (each shown with different color) implicated into a horizontal transmission among 4 patients (samples 1–4; viral titer of 10^4^–10^6^ IU/ml).

HBV evolution and intra-host population structure are frequently monitored using short regions of the HBV genome such as the *S* gene [3]. However, because data on the HBV WG quasispecies are very limited, it is not clear whether quasispecies of short regions adequately reflect intra-host HBV evolution. The distribution of polymorphic sites along the HBV WG quasispecies is shown in Fig. 6. Alignment of all WG sequences from independent samples belonging to different genotypes (B, D, E and G) showed the presence of nucleotide changes occurring throughout the entire genome, with regions of apparent substitution clustering including the RT domain, and the core and spacer regions (Fig. 6a). Furthermore, WG quasispecies from genetically close HBV variants of genotype D identified in patients (samples 1–4) linked by horizontal transmission showed a similar profile (Fig. 6b). These observations suggest that nucleotide variations occur across the entire genome within and between genotypes, and there is no particular short sequence that could be analyzed independently to accurately evaluate viral evolution.

### Diversity of HBV quasispecies population

**Figure 7:**
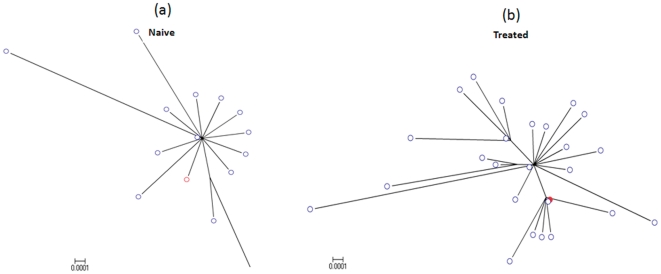
Diversity in naive and treated individuals. Phylogenetic trees of HBV-WG quasispecies (blue) and consensus sequences (red) from: (**a**) a treatment-naïve patient (viral titer −10^3^ IU/ml) and (**b**) a lamivudine-treated patient (viral titer −10^6^ IU/ml).

Quasispecies diversity of lamivudine-naïve and -treated individuals were evaluated. The star-like phylogeny reflects lower genetic diversity of the complete genome in naïve compared to treated subject (Fig. 7). Unlike the naïve case, the consensus sequence of the treated subject was not reflective of the entire viral population. Nucleotide diversity of the treated group (n = 8) was on average 3 fold greater than in the naïve group (n = 8), the difference being significant (p = 0.0194).

## Discussion

Understanding the evolution of HBV genome, and identifying mutations that determine phenotypic variations in different clinical and epidemiological settings are important for management of HBV drug resistance and vaccine escape. In this study, we describe for the first time the use of the EPLD-PCR approach for analyzing viral quasispecies of the HBV genome of different genotypes. WG quasispecies information obtained using this approach is derived from independent DNA molecules rather than from consensus sequence of all intra-host variants, as is the case with deep sequencing approaches [19], [20]. This EPLD-PCR novel approach provides important information for delineating the dynamics of the intra-host viral population and understanding the genetic linkage among genomic sites.

The main features of the EPLD-PCR-based approach to HBV WG quasispecies sequencing are that it: (i) is real-time-based which thereby circumvents the need for post-PCR cloning, (ii) has high sensitivity, (iii) has minimal PCR-prone errors due to the use of high-fidelity enzyme, (iv) uses the same PCR conditions for all sets of PCR primers; (v) has equal sensitivity for all six nested primers, (vi) incorporates tagged nested primers allowing use of only 2 sequence primers, and (vii) is adaptable to automation so reducing cost, hands-on time and probability of cross-contamination.

It is frequently assumed that the end-point limiting-dilution protocol for amplification of individual genetic variants depends on dilution down to a single molecule [26], [27]. Recently, we have shown that this dilution is rather determined by the probability of amplification of only a single molecule from a pool of molecules. The minimal size of the pool is defined by the efficiency of PCR amplification, with less efficient PCR conditions requiring a larger pool of molecules for single-molecule amplification [22]. This finding suggests that, although amplification of long DNA molecules like HBV WG has low probability of being achieved from a single molecule of the HBV genome, a single HBV genome variant can still be amplified from a small pool of DNA molecules at that dilution. Such a principle was applied in this method developed for HBV WG quasispecies analysis.

To improve efficacy of quasispecies detection and sequencing, we devised a second-round PCR using 6 sets of PCR primers for amplification of small overlapping fragments. Theoretically, such amplification protocol may result in assembly of mosaic WG sequences if some of 6 PCR fragments were derived from different HBV variants. However, the experiments conducted in this study showed that there is a very low chance of amplifying a mixture of HBV WG at the end-point. This observation strongly validates the use of 6 PCR fragments in the second-round amplification for HBV WG quasispecies analysis. This is also proof of concept that the amplification of HBV WG at limiting dilution is narrowed down to a single molecule randomly selected from a small pool of DNA molecules, while the direct PCR amplification for consensus sequencing uses a mixture of DNA molecules. Such consideration underlies why consensus sequence-based methods are only capable of detecting substitutions present in viral quasispecies with a prevalence of >20% of the total HBV population [28].

Amplification of WG quasispecies through EPLD appears to have the propensity to recover both the commonly occurring genomes, and those that do not belong to the main lineage. Detection and molecular characterization of the viral population, including minor variants, at the WG level is important for understanding the development of drug resistance. For example, it is known that minority drug-resistant human immunodeficiency virus variants arising during therapy are significantly relevant clinically, as they can quickly grow out when subjected to the selective pressure exerted by the drug to then become dominant, so leading to treatment failure [29], [30]. Many published data have shown that the appearance of resistance mutations may precede the increase in viral load (virological resistance) by several months [31] and could be a prognostic marker for the occurrence of viral breakthrough. However, since WG sequences of intra-host HBV variants are only infrequently available, changes in the genetic structure of the intra-host HBV populations under the selection pressure of therapeutic treatment are usually assessed using subgenomic fragments. A significant conservation of the HBV genome limits genetic variability of subgenomic regions [3], [4], thus preventing the accurate reconstruction of intra-host HBV populations. Fig. 7 shows changes in the genetic composition of the intra-host HBV population after the therapeutic treatment. Although application of drugs inhibiting viral replication in treated patients seems to provide the strongest selection pressure, HBV should be under many other selection pressures related to the natural course of chronic infection such as neutralizing immune responses. Interaction among different selection forces may affect the outcome of therapy and requires a thorough evaluation, which can be achieved through analysis of WG HBV sequences. This analysis is key to the identification of epistatic connectivity among genomic sites [32], and to relating this connectivity to outcomes of therapeutic treatment [5], [33]. Only a limited number of HBV mutations selected during antiviral treatment have been well characterized, and the diagnostic and public health implications of other mutations occurring at the whole genome level need further investigation.

The application of this novel technique to analysis of HBV WG quasispecies is also important for the identification and evaluation of recombination events. HBV recombination has been frequently detected during co-infection of hosts with more than one HBV genotype, presenting an additional source of variability in HBV genome [34], [35]. In the present study, we have tested HBV WG sequences using SIMPLOT for the detection of recombination and for mapping exact recombination points. Bootscan analysis of one study patient indicated that recombination emerged in the core and RT gene. It has been reported that the sites of recombination appeared different in each variant, suggesting that recombinant variants are subjected to selection, and only one or a few variants ultimately become dominant [36]. The ability of EPLD-PCR analysis demonstrated here in identifying co-infection, viral sub-populations and recombination events substantiates the versatility of this approach for HBV WG quasispecies analysis.

A critical observation made in this study was that HBV-WG consensus sequences obtained by direct sequencing of PCR fragments without EPLD are not always identical to the major HBV variants in the intra-host population. In some cases, HBV consensus sequences were found to be different from the closest intra-host HBV variants at 1–2 genomic sites. This difference might be attributed to the complex pool of HBV DNA molecules present in the sample and the stochastic nature of PCR. Variation in amplification of different molecular species from the sample among 6 PCR fragments used in the protocol for consensus sequencing may result in reconstruction of hybrid WG sequences. This is overcome in the EPLD process as the amplification is limited to a single or minimal number of DNA molecules. The observation of the discrepancy between the consensus sequences and intra-host HBV variants indicates that WG consensus sequences should be judiciously used in genetic analysis. Sequence-based studies of HBV WG quasispecies should afford a more accurate assessment of HBV evolution in various clinical and epidemiological settings.

The data presented here project the strategy of amplifying full-length HBV DNA quasispecies with minimal distortion using EPLD approach. Unlike cloning approach where clonal selection is performed after PCR amplification, EPLD-PCR introduces the least PCR errors as DNA is diluted ahead of any amplification process, thereby minimizing template jumping, allelic preference, etc. There may be still concerns about primer bias and/or mismatches with at least some archived HBV sequences which might limit the outcome of their analyses. However, any improvement to the current methodology, such as using more efficient DNA polymerases (phusion DNA polymerase), can be improvised to minimize the PCR-prone errors.

In comparison to ultra-deep sequencing technologies, the EPLD approach generates fewer sequences. However, the EPLD approach readily generates HBV WG sequences. We and others have observed that assembly of such long sequences for individual variants from short reads produced by the ultra-deep sequencing technologies generates extremely challenging computational tasks [14], [21]. Additionally, the EPLD approach does not require any additional equipment besides conventional equipment for PCR and sequencing that is commonly available in many molecular laboratories, nor do the data generated require extensive computational assembly. Thus, the novel EPLD PCR-based technique described here for sequencing of individual HBV complete genome variants from a single DNA molecule is conveniently amenable to HBV WG quasispecies analysis, thereby allowing in-depth characterization of intra-host HBV populations, detection of recombination events and evaluation of epistatic connectivity along the HBV genome that facilitates investigation of genomic co-evolution in clinical and epidemiological settings.

## Materials and Methods

### Study samples

Randomly selected serum samples were collected from patients with acute and chronic HBV infection (n = 38) identified in the course of transmission and surveillance studies carried out by the Centers for Disease Control and Prevention, Atlanta, GA. Informed consent had been obtained from all participants and ethical approvals were granted by the CDC ethics review board. Samples were tested for: antibodies to hepatitis C virus (Ortho HCV ELISA 3.0; Ortho-Clinical Diagnostics); antibodies to HIV-1 (Vironostika HIV-1 MicroELISA System; bioMérieux); and markers of HBV infection, including total antibody to hepatitis B core antigen (anti-HBc; ETI-AB-COREK PLUS; DiaSorin), IgM anti-HBc (ETI-CORE-IgMK PLUS; DiaSorin), hepatitis B surface antigen (HBsAg; HBsAg EIA 2.0; Bio-Rad), hepatitis B surface antibody (anti-HBs; ETI-AB-AUK PLUS; DiaSorin), hepatitis B e antigen (HBeAg; ETI-EBK PLUS; DiaSorin), hepatitis B e antibody (anti-HBe; ETI-AB-EBK PLUS; DiaSorin), and HBV DNA concentration (HBV ASR; Abbott Molecular). Samples from HBsAg-positive patients were analyzed for HBsAg subtype by an enzyme immunoassay [37].

### Nucleic acid extraction

Total nucleic acids were isolated from all serum samples using the robotic Roche MagNA Pure LC system (software version 3.0.11) and the MagNA Pure LC Total Nucleic acid isolation kit (Roche Diagnostics GmbH, Mannheim, Germany), and eluted in 50 ul of lysis buffer according to the manufacturer's instructions.

### Real time PCR

First-round WG PCR was conducted using the GeneAmp® PCR system 9700 (Applied Biosystem) incorporating the Expand High-Fidelity PCR kit (Roche) with the 1823/1801 primer pairs (Table 1). The cycling conditions were: 94°C/3′, 10 cycles of 94°C/20″, 55-45°C/30″ with a step down of 1°C/cycle, and extension 68°C/4′, followed by 35 cycles of 94°C/20″, 45°C/30″, 68°C/4′ increase elongation time 10 sec/cycle up to 7.20 min, followed by a final elongation 68°C/10′. Five ul of the extracted DNA (including direct or log diluted DNA) is used per reaction / 50 ul reaction volume of the 1 st round PCR components which includes 10X buffer containing Mg2+ (5 ul), 10 mM each dNTP mix (1 ul), expand high-fidelity enzyme (0.5 ul of 3.5 U/ul) and primers (1 ul of 50 uM each). For the nested PCR, 1 to 2 ul of the first round product is used. For real-time nested PCR, six sets of overlapping nested primers were used (Table 1, Fig. 1). Primers were designed using multiple standard parameters such as temperature, GC content, hairpin loops, dimer formation, etc., as implemented in PrimerSelect from the Lasergene DNA and Protein analysis software (version 7.0, DNASTAR Inc., Madison, WI.). The PerfeCTa SYBR FastMix chemistry (Quanta BioSciences, Gaithersburg, MD) or Lightcycler Fast start DNA Master^+^ Sybr Green kit (Roche, Applied Sciences, Indianapolis, IN) was used for second-round amplification, and run on LC480 real-time instrument (Roche) or Stratagene Mx3005P instrument (Stratagene, La Jolla, CA). The PCR conditions were: 95°C/10′, 95°C/30″, 55°C/1′, 72°C/32″ for 40 cycles, and followed by melt curve cycle of 95°C/1′, 80°C/30″, 95°C/30″. The nested PCR reaction includes 4 ul of Fast start DNA Master^+^ Sybr mix, 1 ul each of 15 uM primers, 1 st round PCR product 1–2 ul and make up volume to 20 ul.

For consensus WG sequencing, extracted DNA was directly used in the 1^st^ round WG amplification followed by nested PCR of six overlapping fragments. For WG quasispecies analysis, EPLD-PCR was performed using serially diluted DNA [22]. The end-point was found by the ability to amplify the 441-bp amplicon containing the *a* determinant of the S gene (Primer 4) in a nested PCR in 45–50% of the reactions. WG clones were identified by melting curve analysis using the LightCycler^®^ 480 Roche software version 1.5.0.SP3, following which the other five overlapping primer sets encompassing the rest of the genome were amplified by nested real-time PCR. The limiting-dilution that resulted in positivity in three out of six replicates was considered to be the one that was limiting. For each isolate, 96 EPLD amplification reactions were carried out to obtain approximately 48 clones per sample; the exact number varied depending on the viral titer. The setting up of all reactions for PCR, clean up, selection of clones and dilutions of the products for sequencing were performed using Biomek 3000 robotic station (Beckman-Coulter, Completeerton, CA). About 50–60 WG clones were amplified per sample, where the number of clones amplified depends on the viral titer.

### Sequencing

The nested primer sequences contain SP6 or T7 promoter and enhancer sequences at the 5′-ends of the specific primers to enable convenient universal sequencing of all six fragments of the HBV WG. Amplicons were subjected to standard di-deoxy termination sequencing following the recommended by Applied Biosystems BigDyeV3.1 protocol.

### Genetic Analysis

Sequence electrophoregrams were initially analyzed and edited using SeqMan and MegAlign programs from the Lasergene DNA and Protein analysis software (version 7.0, DNASTAR Inc., Madison, WI.). Multiple sequence alignment and evolutionary distances analysis were performed in Accelrys GCG, Version 11.0 (Genetic Computer Group, Accelrys Inc., San Diego, CA). HBV genotypes were classified based on the S sequence, and confirmed with WG sequences by comparing each sequence with published reference sequences from GenBank. The nucleotide diversity [38] of each sample was calculated using the program ARLEQUIN [39].

### Phylogenetic analysis

#### Maximum likelihood trees

The program ModelTest [40] was used to establish the best model of RNA substitution for our HCV data. The GTR model was chosen to create maximum likelihood trees (ML) [41] using the program HyPHY [42]. An initial tree was created using the neighbor-joining approach [43], a search in the tree space was performed using nearest neighbor interchange branch on the tree until no further likelihood score improvements could be made.

### Detection of inter-genotypic recombination

Recombination was examined in the WG sequences using bootscan in SimPlot (ver. 3.5.1) [44]. Reference sequences for HBV genotypes A to H were retrieved from GenBank, and sequences with recombinant fragments were removed from this analysis. In each case, sequence fragments of 200 bases incrementing by 20 bases, with 100 bootstrap replicates, were compared with consensus sequences of the human HBV genotypes.
